# Repurposing the skin to heal the body: Tissue Nanotransfection (TNT) as a therapeutic platform

**DOI:** 10.1007/s44258-026-00084-8

**Published:** 2026-06-01

**Authors:** Ana I. Salazar-Puerta, Kristin Stanford, Natalia Higuita-Castro, Daniel Gallego-Perez

**Affiliations:** 1 Department of Biomedical Engineering, The Ohio State University, Columbus, OH USA; 2 Department of Surgery, The Ohio State University, Columbus, OH USA; 3 Davis Heart and Lung Research Institute, The Ohio State University, Columbus, OH USA; 4 Department of Neurological Surgery, The Ohio State University, Columbus, OH USA; 5 Gene Therapy Institute, The Ohio State University, Columbus, OH USA

**Keywords:** Tissue Nano-Transfection, In situ therapeutic manufacturing, Non-viral gene delivery, Regenerative medicine, Organ-targeted delivery

## Abstract

Tissue Nanotransfection (TNT) is an emerging and transformative platform for non-viral drug and gene delivery that enables highly efficient, precise, and rapid (millisecond-scale) transfer of molecular cargo into solid tissues. Using silicon nanochannels, TNT electrophoretically drives charged payloads (*e.g.,* plasmid DNA, miRNA, etc.) through transient membrane pores created by brief electric pulses. Beyond its capacity for localized delivery, TNT can induce systemic therapeutic effects by repurposing tissues in situ to function as biofactories. Recent studies demonstrate that TNT-engineered skin can be harnessed to release extracellular vesicles (EVs) enriched with delivered molecular cargo and functionalized with targeting ligands, as well as potent metabolites capable of modulating distant tissues. These engineered products can engage in inter-organ communication, extending the biological reach of localized interventions for both therapeutic and fundamental research applications. Moreover, TNT could conceivably be used to generate reprogrammed cells within the skin that migrate to extracutaneous sites, where they may contribute to tissue repair, immune modulation, or other systemic effects. Altogether, this precision-driven and benign delivery approach positions TNT as a transformative non-viral technology capable of converting accessible tissues into programmable bioactive hubs that modulate systemic physiology and disease, offering new avenues for novel therapies and mechanistic discovery.

**Graphical Abstract:**

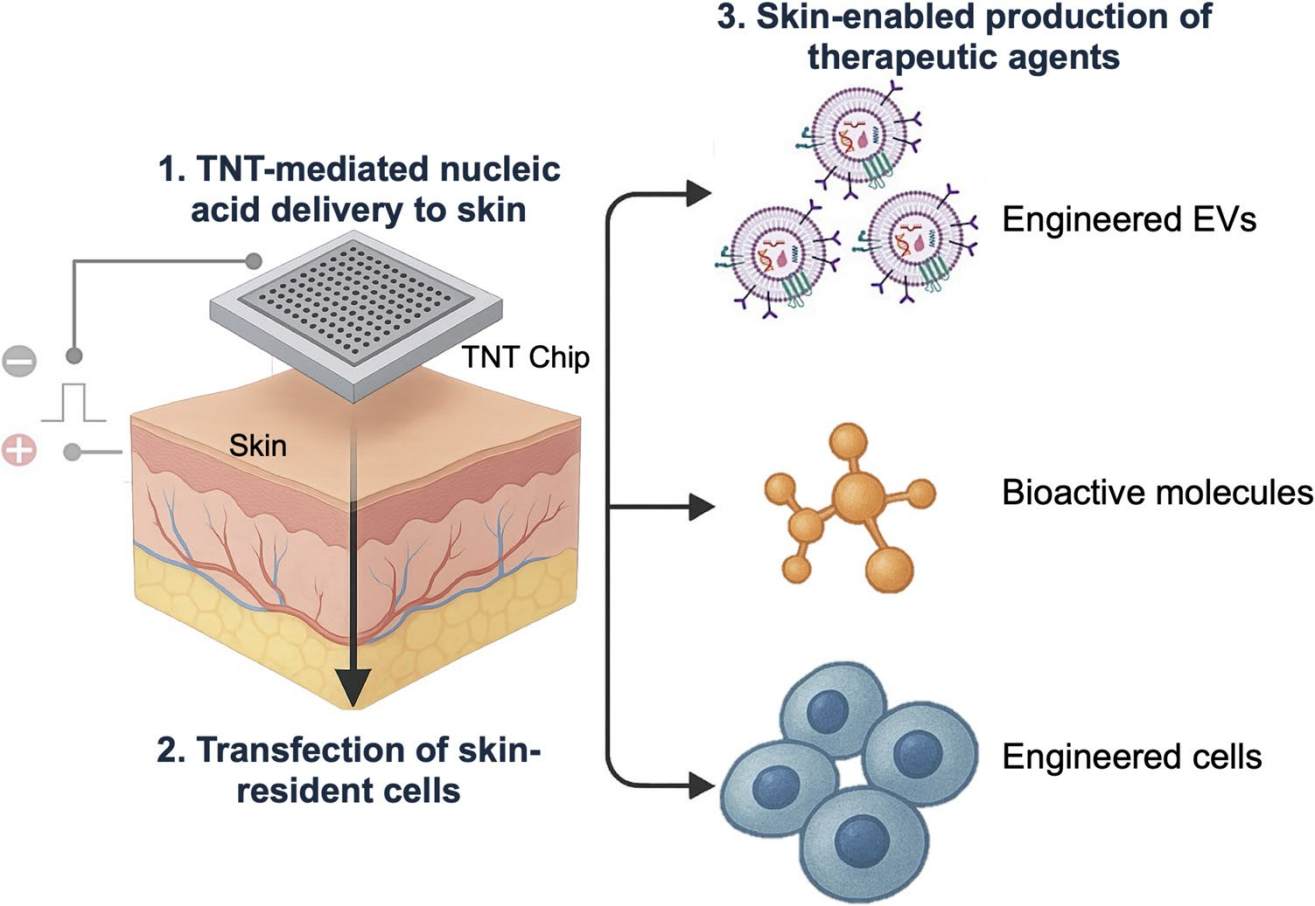

## Highlights

- TNT turns accessible tissues into biofactories that release extracellular vesicles (EVs) and bioactive molecules that influence distant organs.
- Beyond therapy, these capabilities enable fundamental studies of inter-organ communication and systemic molecular effects.
- Future TNT adaptations may generate engineered cells that act beyond the application site, enabling controlled systemic effects.

## Introduction

**Fig. 1:**
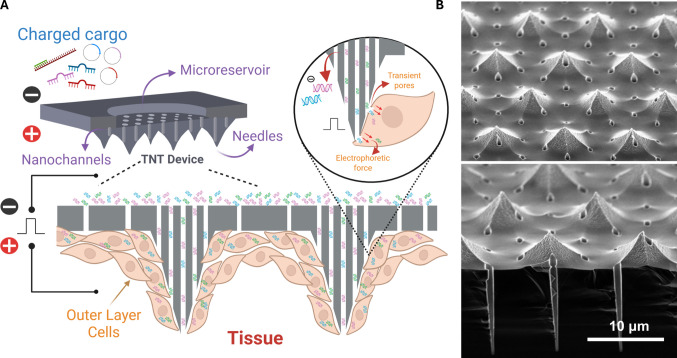
TNT enables non-viral gene delivery to solid tissues. **A** Schematic illustration of the silicon-based TNT chip, highlighting the protruding nanochannels, microneedles, and cargo microreservoirs, and their interaction with the outermost tissue cells in direct contact with the device. Upon application of an electric field, transient pores form on the cell membranes, allowing charged cargo to enter the cells via electrophoretic forces. **B** Scanning electron microscopy (SEM) images showing the platform and nanochannel arrays protruding from the device surface (needle pattern), designed to interface with tissue-resident cells

Tissue nanotransfection (TNT) is an emerging, technology readiness level 4 (TRL4) platform for non-viral drug and gene delivery that enables highly efficient, rapid, and safe cargo deployment into solid tissues [1, 2]. Unlike conventional approaches such as viral vectors, nanoparticles, bulk electroporation (BEP), or ballistic gene delivery (*i.e.,* gene gun), which largely depend on bulk electric fields, particle-mediated ballistic penetration, or stochastic cargo uptake and are often associated with variable efficiency or reduced cell viability, TNT employs silicon nanochannels to transiently porate cell membranes and electrophoretically deliver charged cargo directly into the cytosol within milliseconds (Fig. 1), enabling highly localized and reproducible delivery. Comparative studies with BEP, for example, show that TNT achieves 50–250-fold higher gene expression than BEP while minimizing tissue injury, establishing a superior risk–benefit profile [1].

TNT has been successfully used to deliver a range of nucleic acids, including microRNAs, synthetic oligonucleotides, and plasmid DNA as large as ~ 10 kbp [1, 2]. Cargo transport in TNT is governed by electrophoretic mobility rather than endocytic uptake, such that delivery efficiency largely depends on cargo size, conformation, and net charge. Accordingly, neutral or weakly charged molecules are not well suited for TNT-based delivery unless chemically modified to confer sufficient charge. While plasmid DNA on the order of ~ 10 kbp is deliverable, the upper size limit for TNT-mediated cargo delivery has not yet been systematically defined.

**Fig. 2:**
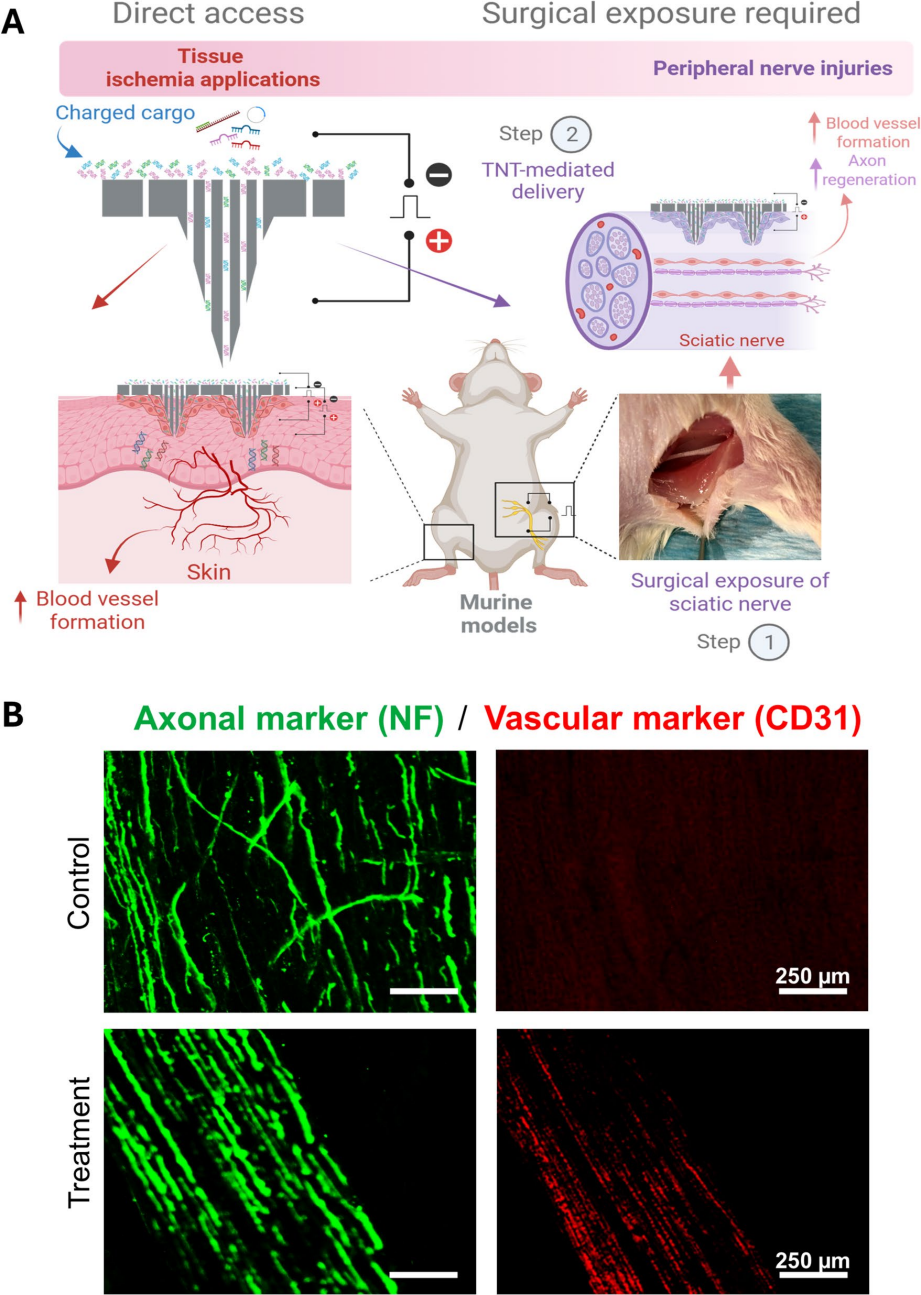
TNT supports localized regenerative interventions. **A** Schematic representation of TNT deployment on skin and peripheral nerves to promote tissue regeneration. Applications include localized controlled stimulation of blood vessel formation via vasculogenic TNT to reverse ischemia and support axonal repair. **B** Histological analysis of longitudinal sections post-injury and TNT treatment reveal that the newly deposited vasculature (CD31, red) provided an aligned scaffolding structure that supported organized axonal regeneration (Neurofilament, green) in the vasculogenic TNT group, in contrast to the disorganized axonal regeneration observed in the control

The skin has naturally been the primary target for TNT development. As the largest and most accessible organ of the body, skin is ideal for both experimental optimization and clinically meaningful applications. Importantly, restricting interventions to the skin offers an inherent safety margin, as the tissue naturally undergoes renewal, treated areas are readily monitored, and, if necessary, can be excised. However, the versatility of TNT has been demonstrated not only across multiple types of cargo but also tissues (*e.g.,* skin and nerve) [1, 3–9]. In these examples, TNT has been applied focally at the site of interest, resulting in spatially restricted gene delivery and biological effects confined to the treated tissue. Such localized applications span diverse preclinical disease models, ranging from ischemic injury to peripheral nerve repair. Cutaneous and perineural applications of TNT have been especially illustrative of its therapeutic breadth. Localized interventions include the delivery of vasculogenic transcription factors to stimulate blood vessel formation through cellular reprogramming to reverse tissue ischemia [1, 4], as well as to stimulate axonal regeneration [7, 10] (Fig. 2). TNT has also been shown to mitigate local wound infections and to induce neurogenic responses within the skin, resulting in improved outcomes in preclinical models of peripheral neuropathy [1, 6]. TNT therefore functions as a broadly adaptable, non-viral delivery system with potential to enable spatially and temporally controlled therapeutic interventions in vivo.

Nevertheless, the transformative potential of TNT is rooted not only in local effects but also in its ability to drive therapeutic responses beyond the site of treatment. TNT can program tissues to release engineered extracellular vesicles (EVs), which are nanosized, lipid bilayer-enclosed particles naturally secreted by all cell types. These vesicles mirror the molecular profile of their donor cells and are loaded with bioactive molecules (nucleic acids, lipids, metabolites, etc.) that mediate intercellular communication [11]. By delivering genetic material into tissues, TNT effectively engineers the resident cells, enabling them to produce EVs with tailored cargo and cell-targeting motifs. This process can also induce the localized production and secretion of therapeutic biomolecules such as peptides and lipids capable of acting systemically.

In addition, a key advantage of TNT is its amenability to redosing and its ability to be administered non-invasively within milliseconds, making it suitable for outpatient care and potentially adaptable to simplified delivery paradigms akin to vaccination or even self-administration approaches, such as epinephrine autoinjectors or insulin pens. Taken together, these features position TNT as an efficient, safe, and versatile platform that combines the accessibility of localized delivery with the ability to drive systemic therapeutic responses. The central thesis of this Perspective is that surface-level TNT should be considered a gateway to non-invasive systemic and organ-specific therapies, positioning the skin not only as a treatment site, but as a bioreactor capable of processing and manufacturing therapeutic cues under immune surveillance to address conditions well beyond the skin itself.

## Why go beyond the skin?

Although TNT can be directly applied to select extracutaneous tissues through surgical or interventional access, such approaches are inherently invasive and constrain its broad clinical utility. However, the ability of TNT to leverage or repurpose the skin as a ‘dispatch’ center of therapeutics for extracutaneous targeting, offers the possibility of reaching such compartments through non-invasive and simpler-to-implement cutaneous interventions. Thus, a compelling rationale emerges for harnessing the skin itself as a gateway for broader, whole-body effects.

**Fig. 3:**
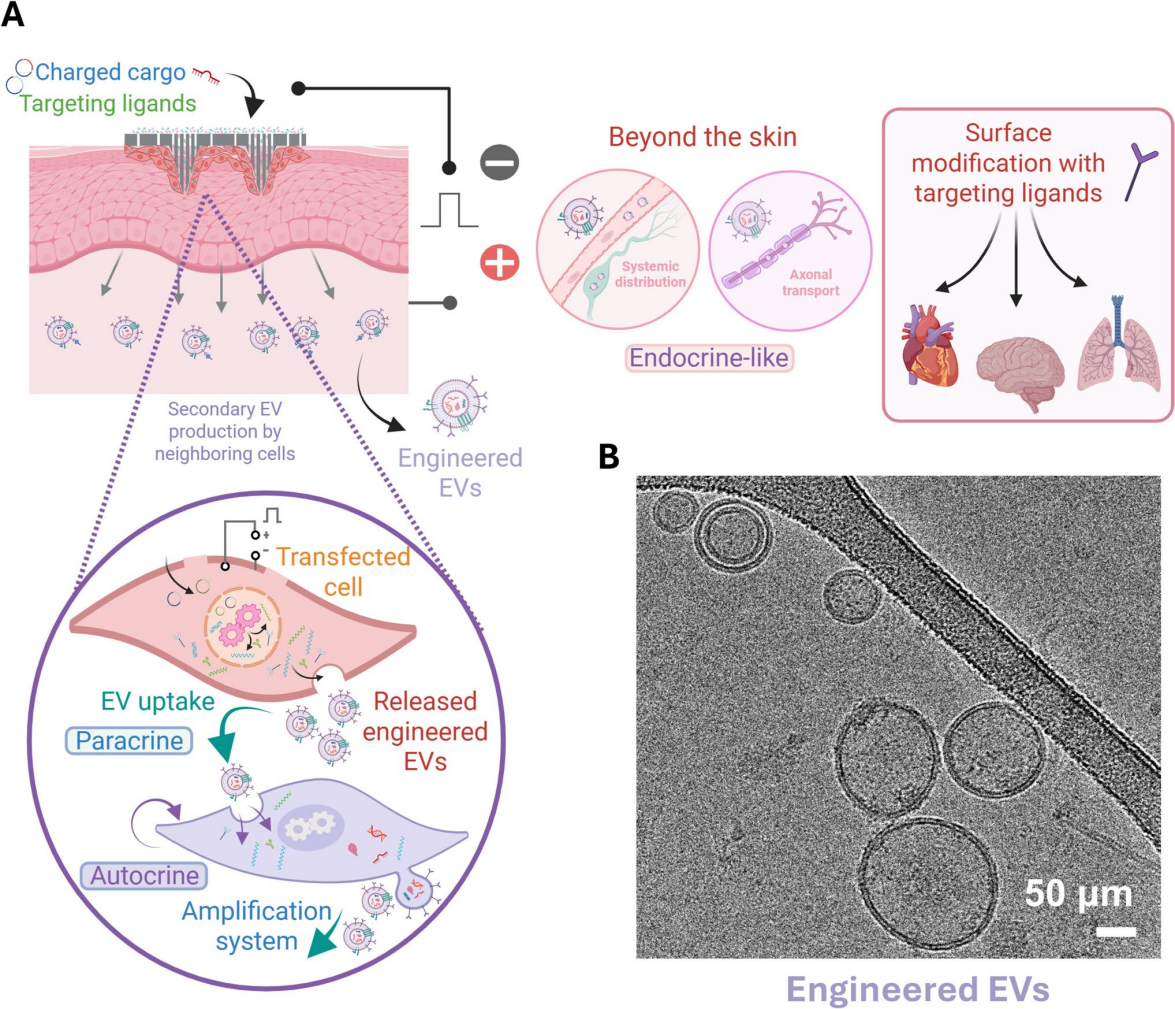
TNT interventions mediate the production of engineered EVs as an amplification system. (**A**) Schematic depicting how TNT-treated tissue facilitates the production of engineered EVs carrying the introduced cargo. These vesicles can then act locally on the same cells (autocrine), spread to adjacent or deeper tissue layers beyond the TNT-treated surface (paracrine), or reach distant organs through systemic circulation (endocrine-like). This amplification and propagation of signals extend the therapeutic reach of the localized TNT intervention. **B** Cryo-electron micrograph of engineered EVs revealed an intact structure

Through TNT-mediated delivery of plasmid DNA, for example, skin-resident cells can be programmed not only to express defined molecular cargo destined for EVs, including nucleic acids and proteins, but also to overexpress specific membrane or surface proteins with organ- or tissue-selective binding properties. These membrane-associated proteins can be incorporated into the EV surface during vesicle biogenesis, thereby functionalizing EVs with targeting ligands that enable preferential uptake by distant cell populations (Fig. 3). In this manner, TNT provides a means to simultaneously control EV cargo composition and surface identity, facilitating targeted, systemically active EV-based therapies initiated from a localized skin application. This strategy addresses a major bottleneck in the EV field, which is the current need for labor-intensive isolation and purification processes prior to clinical use [12]. By generating therapeutic EVs directly in vivo, TNT obviates these barriers and creates a renewable source of targeted vesicles. Additional safety layers, such as cell-type-specific promoters, could ensure that only selected cell populations produce the engineered products, or that such engineered products (*e.g.,* EVs) only act on select cells, further refining control over systemic delivery.

Beyond EVs, TNT can be harnessed to drive the production of soluble factors with broad systemic impact. These include hormones, cytokines, lipokines, and other signaling molecules whose physiological roles are naturally widespread. By repurposing accessible skin cells to express and release defined amounts of such molecules, TNT could enable sustained therapeutic modulation of metabolism, immune function, or tissue repair, among others. For example, upregulation of exercise-mimetic lipokines could provide benefits to patients unable to engage in physical activity, while inducible cytokine production could bolster immunity or promote graft tolerance in transplantation.

Preclinical studies have already demonstrated the potential of this paradigm. In one notable example, cutaneous TNT was used to modulate breast tumor progression through the generation of engineered EVs emanating from TNT-treated skin [5]. In this study, TNT-programmed skin cells produced EVs decorated with intercellular adhesion molecule-1 (ICAM-1), enabling preferential accumulation within the myeloid cell compartment of the breast tumor. Once localized to the tumor niche, these EVs mediated metabolic reprogramming of myeloid cells, shifting them from an immunosuppressive, anti-inflammatory state toward a pro-inflammatory phenotype. This reprogramming restored immune surveillance within the tumor microenvironment and suppressed tumor progression, demonstrating how TNT-driven EV engineering can exert therapeutic effects at distant, extracutaneous sites. To date, this remains the only reported study documenting TNT-enabled production of engineered EVs for extracutaneous tissue therapy, highlighting both the feasibility of this approach and the substantial opportunity to expand TNT-based interventions toward the generation of EVs bearing diverse targeting moieties for other organs and disease contexts (Fig. 3). In parallel, alternative TNT-enabled systemic strategies have been described, including the use of skin as a metabolic factory to upregulate the lipokine 12,13-diHOME, which exerts endocrine-like effects across multiple organs to counteract high-fat-diet-induced cardiometabolic dysfunction and improve cardiac performance in preclinical models of metabolic dysfunction [8] and aging [9]. Together, these examples illustrate how localized TNT interventions can be harnessed to drive EV-mediated targeting or bioactive metabolite production, thereby impacting whole-body physiology and disease far beyond the site of treatment. By extending its reach beyond the skin, TNT has the potential to bridge local interventions with systemic or organ-targeted therapies, opening a new frontier of therapies previously considered unattainable.

## Pathways to whole-body impact

Several routes may enable TNT-generated products to reach extracutaneous targets. Engineered factors could access systemic circulation or lymphatic drainage, providing pathways for distribution across the body. Alternatively, tissue-resident immune cells exposed to the TNT-treated patch could carry therapeutic cargo to distant sites as they migrate. Another intriguing possibility is axonal transport. Given the dense innervation of skin, molecules or vesicles produced at the cutaneous surface could potentially be taken up by nerve endings and shuttled to other sites, including the central nervous system, providing a non-invasive means of therapeutic delivery to difficult-to-reach locations [13].

In addition to biochemical dissemination, TNT also holds promise for generating therapeutic cells with disease-homing properties through in vivo cell engineering or reprogramming. For instance, TNT could induce the formation of neural progenitor cells, which are known to migrate toward gliomas and other brain lesions [14–19]. Similarly, reprogramming to endothelial or mesenchymal progenitor phenotypes could produce cells that preferentially home to sites of ischemia, injury, or disease [20–23]. These cells could then act as autonomous therapeutic vectors [24, 25], extending the impact of a cutaneous TNT intervention to otherwise inaccessible tissues. An important consideration, however, is the extent to which these induced cells can maintain their phenotype during extracutaneous dissemination. Phenotypic stability will be a critical determinant of their migratory capacity, homing specificity, and ability to exert sustained therapeutic effects.

Whether through engineered EVs, secreted molecules, or disease-homing cells, TNT offers multiple mechanistic routes to transform a local intervention into a systemic or organ-targeted therapeutic strategy, bridging the gap between localized delivery and whole-body impact.

## Challenges and unknows

While TNT holds transformative potential, ongoing de-risking efforts will be key to enabling its broader clinical translation. Biologically, one key goal is achieving precise biodistribution when TNT is used to generate systemic therapeutics. Off-target effects in non-diseased “pass-through” tissues may limit efficacy or cause toxicity. Engineering EVs with defined surface decorations and employing cell- or tissue-specific promoters could mitigate this risk by enhancing targeting specificity while reducing nonspecific interactions with clearance organs. Another potential challenge lies in immune compatibility. Although TNT’s non-viral nature reduces immunogenicity and enables safe redosing, unanticipated immune responses to reprogrammed cells or novel proteins remain possible. One mitigating factor is that in situ production occurs under constant immune surveillance, which may improve tolerability [26–29]. Importantly, cutaneous TNT also offers built-in safeguards, given that the magnitude of systemic effects can be controlled through the frequency or size of skin patches treated, and treated sites can be readily monitored or excised, if needed.

In addition, a key open question for in situ therapeutic EV production via TNT is the magnitude and temporal profile of EV output relative to ex vivo-manufactured EV products. At present, quantitative comparisons are challenging, as EV biogenesis and release dynamics of cells engineered in vivo may differ substantially from those of cells expanded and conditioned in vitro, both in terms of total EV yield and production kinetics. Moreover, TNT-modified cells in their native tissue context are likely to release EVs in a sustained or regulated manner, in contrast to the bolus administration typical of ex vivo-derived EV therapies. Such differences in release kinetics, tissue localization, and cumulative exposure could meaningfully influence biodistribution, target engagement, and therapeutic efficacy, even if absolute EV numbers are lower than those achieved with exogenous dosing. In addition, the method used to induce EV production (*e.g.,* TNT, lipid nanoparticles, conventional electroporation, or other gene transfer approaches) may itself shape cellular stress responses, transcriptional programs, and vesicle biogenesis pathways, thereby affecting EV quantity, composition, and functional potency. Systematic studies that jointly evaluate engineering modality, EV production dynamics, and functional dose equivalence between in situ and ex vivo approaches will therefore be essential to define optimal therapeutic paradigms and to determine when continuous, localized EV generation may offer advantages over intermittent systemic delivery.

Another unresolved challenge is the ability to precisely control and monitor the biological half-life, circulating concentration, and functional exposure of TNT-derived therapeutic products. At present, the pharmacokinetics of these agents when produced in situ, particularly their release rates, systemic persistence, and clearance pathways, remain poorly defined. As a result, strategies for maintaining TNT-derived therapeutics within a defined therapeutic window, while minimizing the risk of systemic overexposure or off-target toxicity, have not yet been established. Addressing this limitation will require the development of new control and monitoring capabilities, such as inducible or self-limiting genetic programs, tissue-specific or feedback-regulated expression systems, and noninvasive biomarkers or imaging approaches to track circulating levels and biodistribution in real time. Systematic pharmacokinetic and pharmacodynamic studies will therefore be essential to translate TNT-based in situ production platforms into safe, predictable, and clinically controllable therapeutic modalities.

Engineering challenges present a second layer of complexity. Device safety must be rigorously validated, as TNT relies on controlled electrical pulses to deploy cargo into tissue. While early preclinical studies suggest a favorable safety profile, electric field exposure may be contraindicated in specific patient populations, including individuals with cardiac pacemakers, implantable cardioverter-defibrillators, deep brain stimulators, or other electrically sensitive implants. Additional contraindications may also emerge depending on field strength, pulse duration, and anatomical site of application. Addressing these risks will require systematic evaluation of delivery parameters alongside the development of mitigation strategies, such as localized field confinement, real-time impedance monitoring, adaptive pulse modulation, and device safeguards designed to minimize stray currents or unintended coupling with implanted hardware.

Moreover, delivery protocols will need to account for patient-specific variables, including age, sex, skin thickness, tissue conductivity, and anatomical location, to ensure both safety and reproducibility across diverse populations. This could potentially be done through the incorporation of real-time or near-real-time tissue sensing, such as impedance-based measurements performed immediately before or during TNT application, to estimate individualized electrical properties of the skin and underlying tissue. These measurements could then be used to automatically or semi-automatically calibrate key electric pulse parameters, including voltage amplitude, pulse duration, and waveform, enabling consistent local field exposure and transfection efficiency while maintaining defined safety thresholds. Collectively, these considerations underscore the need for integrated device engineering, adaptive control systems, and clinical validation efforts to establish TNT protocols that are broadly deployable while maintaining robust safety margins across heterogeneous patient populations.

Translational and regulatory hurdles are also important to consider. The most pressing is safety testing, particularly for applications where repeated interventions are needed. However, TNT may benefit from precedents set by other electric field-based medical technologies already in clinical use, including neuromodulation devices [30], pacemakers [31], defibrillators [32], tumor treating fields [33], electrochemotherapy [34], and wound healing stimulators [35]. Leveraging this regulatory history could ease the path forward.

Together, these challenges underscore the importance of carefully staged development, balancing TNT’s bold potential with realistic pathways toward safe, ethical, and equitable translation.

## Vision for the future

TNT is likely to follow a staged trajectory from bench to bedside and ultimately beyond the skin. In the near term, refinements will center on device safety, reproducibility, and practical deployment, with early applications in localized settings such as wound healing and limb ischemia. As the platform matures, early-phase clinical testing could establish feasibility in cutaneous and peripheral tissues, while TNT also evolves into a broadly adopted research tool for in vivo gene delivery and therapeutic discovery. With further advances, cutaneous TNT may demonstrate systemic benefits mediated by paracrine or endocrine-like factors and engineered EVs.

The transformative therapies that TNT could enable are striking. Cutaneous TNT may be programmed to drive systemic vascular regeneration, endocrine and metabolic control, and the production of brain-targeted therapeutics capable of functionally crossing or bypassing the blood–brain barrier (BBB). In this context, TNT-engineered skin cells could serve as a localized source of BBB-penetrant proteins, peptides, or EVs endowed with intrinsic or engineered brain-homing properties, such as ligand-mediated transcytosis pathways or disease-responsive uptake mechanisms [36–39]. EVs in particular have been shown to traverse the BBB under both physiological and pathological conditions and can be engineered to enhance CNS targeting through surface display of targeting peptides or membrane proteins [40–42]. Sustained, endogenous production of such agents from TNT-modified skin may therefore enable continuous CNS exposure without the need for repeated systemic dosing or invasive intracranial delivery.

Beyond neurotherapeutics, TNT could promote immune tolerance in transplantation, support “infusion-free” cancer immunotherapies through in situ immune cell reprogramming, and modulate systemic immunity more broadly. In parallel, TNT could enable non-traditional applications such as localized enhancement of skin biology through controlled production of extracellular matrix components (*e.g.,* collagen or hyaluronic acid) or other bioactive molecules. Collectively, these possibilities extend TNT’s conceptual scope beyond conventional therapeutic paradigms, highlighting its potential as a programmable biointerface with applications spanning regenerative medicine, immunomodulation, and exploratory cosmetic science.

While the skin provides a convenient and safe entry point for TNT, a parallel track of innovation lies in adapting the technology for direct use in internal organs and tissues. Future applications could leverage minimally invasive surgical platforms, such as catheter-based or laparoscopic approaches, to deliver TNT to sites like muscle, heart, or mucosal surfaces. Direct application to these tissues would not only improve the precision of deep tissue targeting but also open opportunities for those tissues themselves to act as therapeutic factories. For instance, TNT-modified internal tissues could be programmed to produce engineered EVs carrying tailored molecular cargo, enabling propagation of therapeutics beyond the treated site without the need for purification. Similarly, TNT could drive the production of soluble signaling factors that exert localized effects, diffuse into deeper compartments, or be designed to selectively act on adjacent tissues. By bypassing pass-through and clearance organs, such strategies could minimize off-target exposure while maximizing therapeutic efficacy. Together, these future directions highlight how TNT could evolve from a skin-focused technology into a versatile platform for organ-specific and systemic therapies.

## Conclusion

TNT is emerging as a dual-use platform with the capacity to impact both localized and systemic disease settings. Its accessibility through the skin provides unique safety advantages, as treated areas can be easily monitored, redosed, or even excised if necessary. On the other hand, its ability to generate signals, EVs, or engineered/reprogrammed cells with broader reach opens the door to whole-body therapeutic strategies. Realizing this potential will require interdisciplinary collaboration between engineers working across biomedical, electrical, chemical, and materials fields to refine device design; biologists to tailor interventions for specific tissues and pathways; and clinicians to translate these insights into real-world applications across different conditions.

## Data Availability

Not applicable.
